# Lyme disease in companion animals: an updated state-of-art and current situation in Portugal

**DOI:** 10.1007/s11259-024-10532-8

**Published:** 2024-09-11

**Authors:** Rita Picado, Catarina Jota Baptista, André Meneses, Sabrina Legatti, Joana Fonseca, Adriana Belas

**Affiliations:** 1 Faculty of Veterinary Medicine, Lusófona University- Lisbon University Centre, Campo Grande 376, Lisbon, 1749-024 Portugal; 2 Egas Moniz Center for Interdisciplinary Research (CiiEM), Egas Moniz School of Health & Science, Almada, Portugal; 3 Centre for the Research and Technology of Agro-Enviromental and Biological Sciences (CITAB- Inov4Agro), University of Trás-os-Montes and Alto Douro, Vila Real, Portugal; 4 Animal and Veterinary Research Center (CECAV), Lusófona University- Lisbon University Centre, Lisbon, Portugal; 5 I-MVET- Research in Veterinary Medicine, Faculty of Veterinary Medicine, Lusófona University- Lisbon University Centre, Lisbon, Portugal; 6 MED-Mediterranean Institute for Agriculture, Environment and Development, Universidade de Évora, Évora, Portugal; 7 School of Health, Protection and Animal Welfare, Polytechnic Institute of Lusofonia (IPLUSO), Lisbon, Portugal

**Keywords:** *Borrelia burgdorferi*, Borreliosis, Pets, Tick, Vector-borne disease, Zoonosis

## Abstract

Lyme disease (LD) is a globally distributed zoonotic multisystemic condition caused by gram-negative spirochete bacteria of the *Borrelia burgdorferi* complex, transmitted through tick bites. Research on LD in domestic animals in Portugal is limited, potentially leading to underestimating its prevalence. This disease affects many species, including humans, making it a critical public health issue. In domestic animals, LD often presents subclinically or with non-specific clinical signs, complicating its diagnosis. Nevertheless, veterinarians should always consider LD in cases with a history of tick exposure and compatible clinical signs. Diagnostic confirmation can be achieved through serological and other complementary tests. Treatment involves eradicating the bacterial infection and managing clinical signs using a combination of antibiotics, analgesics, anti-inflammatories, and other medications. Effective prevention primarily relies on tick control measures. This review aims to provide an up-to-date state-of-the-art LD, particularly in Portugal.

## Introduction

Lyme disease (LD), or borreliosis, is a zoonotic disease, caused by *Borrelia burgdorferi* sensu lato, that has a worldwide distribution. It is a multisystemic disease whose clinical signs can be very unspecific, making it hard to diagnose (Baneth et al. 2016; Camire et al. 2021; Milkovičová et al. 2023). At least five genospecies are known to cause LD in humans: *Borrelia afzelii*,* Borrelia bavariensis*,* Borrelia burgdorferi* sensu stricto, *Borrelia garinii* and *Borrelia spielmanii* (Stanek et al. 2012; Steere et al. 2016; Eisen 2020). Of these, *B. afzelii*,* B. burgdorferi* s. s., and *B. garinni* are considered pathogenic in dogs (Gatellet et al. 2019; ESCCAP 2023).

Lyme disease can affect a wide variety of domestic (mainly dogs and horses), wild animals and humans, having an important public health importance and reservoirs hosts can acquire this infection when exposed to infected ticks (Parry 2016; Kassab et al. 2020). Cat infection has also been reported, especially in outdoor cats, although there is less information about its specific prevalence, clinical signs and treatment (Pantchev et al. 2016; ESCCAP 2023).

Borreliosis is generally considered one of the most prevalent vector-borne diseases in Europe and North America in humans and dogs (Garrido and Borges-Costa 2018; Camire et al. 2021). In Europe, several pathogenic species of *B. burgdorferi* s. l. can be found. *Borrelia burgdorferi* s. s. is more common in Western Europe; while *Borrelia lusitaniae* is more frequent in Southern Europe, including the Mediterranean area(Mannelli et al. 2012).

In Portugal, LD cases must be reported for humans since 1999, but not for animals (Mannelli et al. 2012). According to INE (2020), in 2017, 20 human cases of borreliosis were reported in Portugal (seven from the north, eight from the centre, and four from Lisbon regions). Due to the biology and epidemiology of this disease, it is difficult to evaluate the real impact of this disease in the country. Moreover, there is a lack of data regarding its actual prevalence. In Portugal, the most prevalent genospecies in ticks is *B. lusitaniae*. However, *B. afzelii*,* B. garinii*, *B. burgdorferi* s. s., *Borrelia valaisiana* and *Borrelia turdi* have also been reported (Nunes et al. 2016; Núncio and Carvalho 2019).

Therefore, this review aims to summarize the information available on biology, epidemiology, clinical presentation and public health aspects of LD in companion animals, focusing on its current situation in Portugal.

## Methodology

This search was carried out using Google Scholar^®^ and Pubmed^®^, with the following keywords: Lyme disease; borreliosis; Portugal; dogs; cats; human. Journal articles, book chapters, and dissertations, published between 2000 and 2023 were included in this study. Moreover, official organization web pages, such as European Scientific Counsel Companion Animal Parasites (ESCCAP), Centers for Disease Control and Prevention (CDC), Companion Animal Parasite Council (CAPC), World Organization for Animal Health (WOAH), Instituto Nacional de Engenharia (INE), World Health Organization (WHO) and European Centre for Disease Prevention and Control (ECDC) were also included. Preference was given to searching for the most up-to-date evidence and Portuguese data.

## Biology, transmission and pathogenesis

Lyme Disease is a vector-borne disease caused by gram-negative spirochete bacteria of the *B. burgdorferi* complex. This zoonotic disease affects multiple domestic animals, wild animals and humans (Parry 2016; Kassab et al. 2020; ESCCAP 2023). *Borrelia garinii*, *B. turdii* and *B. valaisiana* are primarily associated with birds, allowing transmission to long distances; while *B. azfelii* and *B. burgdorferi* s. s. are associated with mammals and *B. lusitaniae* with lizards (Kurtenbach et al. 2002; Norte et al. 2015). Interestingly, certain dog breeds appear to have a predisposition for infection. Adaszek et al. (2022), stated that Bernese Mountain dogs often test positive for antibodies against *B. burgdorferi*, indicating a higher susceptibility to infection of a hereditary infection.

Different tick species from the Ixodidae family may work as vectors of this disease (Margos et al. 2020), including *Ixodes ricinus* and *Ixodes persulcatus* (the main vectors in Europe and Asia), *Ixodes scapularis* and *Ixodes pacificus* (the main vectors in North America/USA), and *Ixodes hexagonus* (Stanek et al. 2012; Steere et al. 2016; Rubio and Seixas 2017). Tick larvae, nymphs and adults can become infected with these bacteria when they feed on infected hosts. For infection to occur, at least 16 to 24 h of blood meal are required (ESCCAP 2023). Vertical transmission between female dogs and puppies is also possible but uncommon (Seixas et al. 2011).

## Public health importance

Vector-borne diseases pose an important threat to public health as they are responsible for more than 17% of all infectious diseases, causing more than 700,000 human deaths annually. They are becoming more and more documented globally (Alho et al. 2016; WHO 2020).

Currently, LD incidence in humans varies between 0.04 and 0.4 cases per 100.000 in Portugal (Departamento de Doenças Infeciosas do Centro de estudos de Vetores e Doenças Infeciosas Doutor Francisco Cambournac 2023).

Tick infestations are typically associated with rural areas. Roome et al. (2022), stated that people living in rural regions of the Northeastern United States, had a 33.6% higher risk of having LD compared to the urban population. However, ticks are expanding their geographical distribution due to animal migration, climate change, globalization, population growth, travel tendencies, changes in the habitat/landscapes, increasing humans’ exposure to ticks, even in urban and suburban habitats (Uspensky 2014; Rizzoli et al. 2014). In addition, an increase in outdoor activities and ongoing human exploitation of environmental resources have also increased different human-vector interactions (Dantas-Torres et al. 2012; Kilpatrick et al. 2017; Vrhovec et al. 2017; Rosà et al. 2018; Alcon-Chino and De-Simone 2022).

Cats and dogs are significant reservoirs of tick-borne diseases. Thus, preventing LD in humans and pets requires a One Health approach (Day 2011; Mencke 2013). According to published data, pet owners are more likely to have an LD diagnosis than people who do not have pets. Dogs can increase their owners’ risk of getting LD by bringing ticks home (Smith et al. 2012; Bowser and Anderson 2018; Herrin et al. 2018). Dog-to-human transmission increases as the number of dogs a person owns grows (Andersson et al. 2017).

Additionally, dogs may not show clinical signs of disease and can act as “silent reservoirs”, allowing for discreet and prolonged transmission to other animals and humans (Alho et al. 2016). On the other hand, cats have been associated with a higher risk of LD. It has been suggested that cats are at higher risk due to lower usage of anti-parasitic drugs compared to dogs, such as tick-preventive collars, and less frequent visual checks by their owners for these parasites. While screening cats for ticks may be more challenging, effective tick-checking among dog owners may have reduced the increased risk for LD associated with dog ownership in some populations (Roome et al. 2022). Nevertheless, in certain geographic regions, cats often have the freedom to come and go from our homes, bringing ticks with them. Moreover, they frequently show a higher predatory behavior, especially for small mammals, such as rodents, which are important reservoirs of ticks and *Borrelia* spp. (Roome et al. 2022).

Therefore, it is crucial to educate the pet owners to use effective preventive measures to prevent tick infection (in both cats and dogs). Extra attention should be given to pets during warmer seasons in both rural and urban areas.

Since companion animals are in close contact with their owners, information regarding the potential risk for the human population can be obtained by collecting ticks from pets and screening them for tick-borne diseases (Liu et al. 2019; Milkovičová et al. 2023). Research has shown that the identification of *B. burgdorferi* directly in dogs or in their ticks is associated with the prevalence of LD in humans in the corresponding areas (Little et al. 2010, 2021; Hendricks et al. 2017; Liu et al. 2019). The same principle applies to the recently confirmed human pathogenic tick-borne relapsing fever spirochete, *Borrelia miyamotoi*, which affects humans and causes *B. miyamotoi* disease. This bacterium is transmitted like *B. burgdorferi*, i.e., through tick bites (*I. ricinus* complex), and as of now is not known to cause disease in dogs and cats. However, systematic surveillance of ticks on domestic animals could be beneficial in assessing human exposure to infected ticks in urban areas (Liberska et al. 2023).

Healthcare professionals must make sure that patients with rash, febrile symptoms, and any possible vector exposure are evaluated for vector-borne infections and actively teach patients about the dangers of vector-borne illnesses and how to prevent them (Lee Werner et al. 2019).

## Epidemiology

Borreliosis is a disease with a worldwide distribution. It is considered the most common tick-borne disease in the temperate areas of the Northern Hemisphere, and it is considered to be endemic in Central and Eastern Europe, North America and Eastern Asia (Bhate and Schwartz 2011; Kilpatrick et al. 2017; Sykes and Makiello 2017; Kassab et al. 2020). Globally, borreliosis affects 0.3 to 0.5 million people annually in the Northern Hemisphere, and only climate change will likely cause a 20% increase in LD incidence in the USA over the next ten years (Kowalec et al. 2017; Eisen 2020). Despite that, there are few published data regarding the incidence and prevalence of LD. However, medical authorities suggest that the number of cases is higher than reported (Cook and Puri 2020). The general distribution of LD is proportional to the geographical distribution of the vectors and reservoirs that are essential to the transmission of these bacteria (Margos et al. 2011).

Some authors believe that the occurrence of this disease is progressively increasing and spreading across Europe (Sykes and Makiello 2017). However, there is no regular surveillance system for LD in companion animals. Therefore, the prevalence of this disease is often inaccurate (Milkovičová et al. 2023). Moreover, free-ranging canines not receiving veterinary care have a smaller chance of being tested and considered in these databases. Additionally, there are fewer diagnostic methods available for cats. Their grooming habits and mainly indoor lifestyle suggest that they have fewer vector-borne diseases than dogs (Qurollo 2019).

Miró et al. (2022) mapped the distribution and seropositivity of dogs for specific canine vector-borne diseases using data from enzyme-linked immunosorbent assay (ELISA) tests in Europe from 2016 to 2020. The presence of *B. burgdorferi* antibodies was mainly concentrated in Northern and Eastern Europe. Higher rates (> 5%) were found in Austria, Czech Republic, Estonia, Finland, Germany, Lithuania, Netherlands, Norway, Poland, Slovenia, Sweden and Switzerland, while lower rates (< 1%) were registered in Andorra, Croatia, Greece, Hungary, Italy, Malta, Portugal, Romania and Spain.

There is limited information on LD in cats. To our knowledge, only three European studies have reported infection with *B. burgdorferi* s. l. in cats. Shaw et al. (2005) reported infection in two cats from the United UK, Tørnqvist-Johnsen et al. (2020) reported infection in another two cats from the UK, and Pantchev et al. (2016) reported infection in 6 cats from Germany and other European countries.

In the northern hemisphere, including western Europe, *B. burgdorferi* s. l. is transmitted mainly by *I. ricinus* and Portugal is considered an endemic country for this vector (Galluzzo et al. 2020; ECDC 2023).

**Fig. 1:**
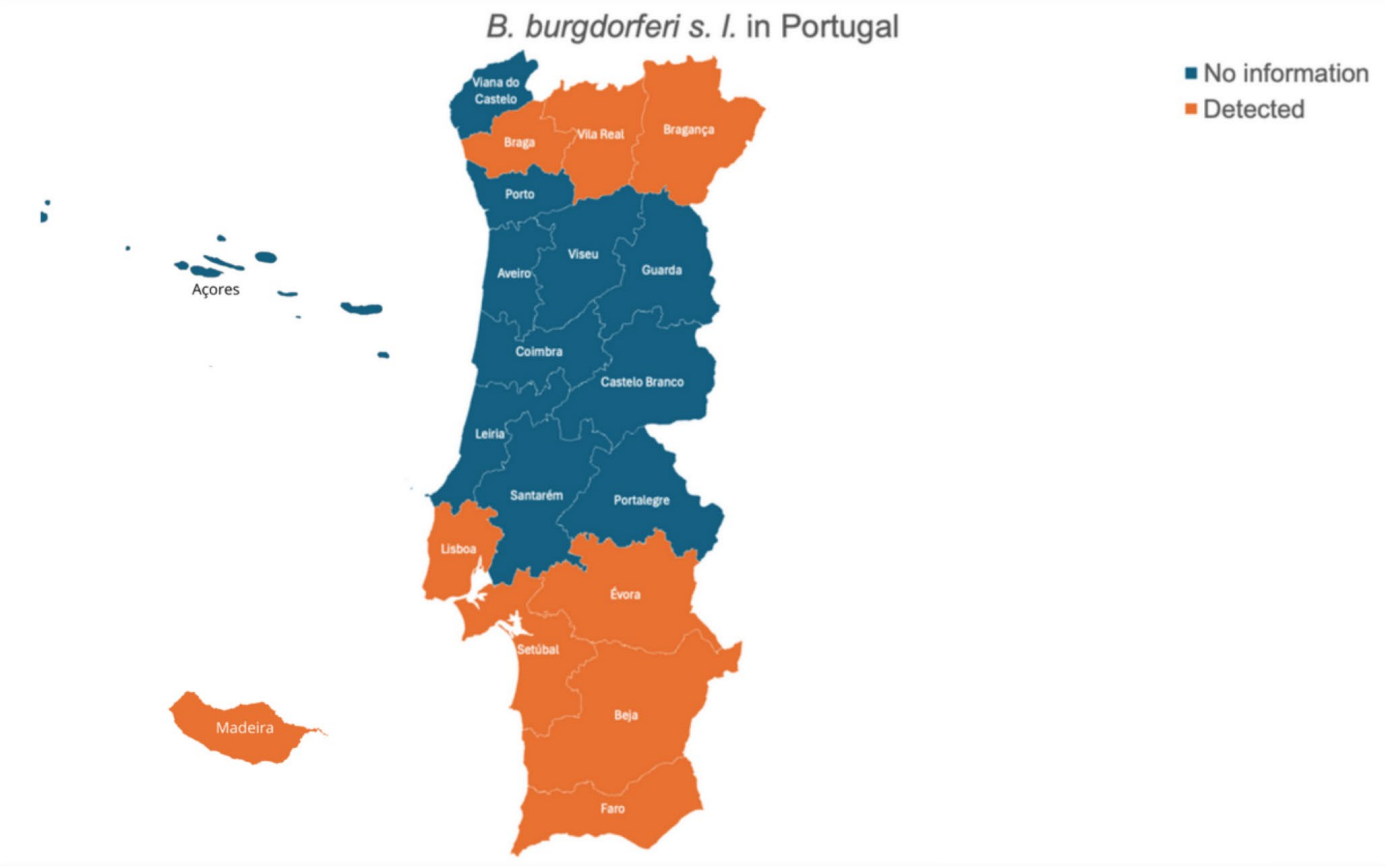
*B. burgdorferi* s. l. in Portugal

Portugal has several endemic vector-borne diseases, primarily attributed to the country’s temperate Mediterranean climate, which promotes vector production and survival (Alho et al. 2016). The studies regarding borreliosis in dogs, cats and ticks in Portugal are summarized in Table 1; Fig. 1.

**Table 1:** Recent studies regarding Lyme disease in Portugal

N	Location	Sample	Study period	Method	Results	Reference
94 military working dogs	Aveiro, Beja, Leiria, Lisbon, Setúbal, Azores and Madeira	Blood	-	Nested PCR	8.5% positive for *B. burgdorferi* s. l. (3.2% *B. afzelli*) in Lisboa, Setúbal, Beja and Madeira	Alho et al. 2016
1010 dogs from veterinary medical centers and shelters	Lisbon, Setúbal, Évora, Beja and Faro	Blood	December 2011 to April 2014	Nested PCR	0.8% positive for *B. burgdorferi* s. l.	Maia et al. 2015
557 healthy dogs and 628 CVBD-suspected dogs from Veterinary medical centers	North, Center, Alentejo, Lisbon, Algarve, Azores and Madeira	Blood	October 2010 to April 2011	Snap 4DX	0.2% positive for *B. burgdorferi* s. l. (healthy dogs) and 0.5% (CVBD-suspected) from Alentejo, North, Center, and Lisbon	Cardoso et al. 2012
400 dogs	Algarve	Blood	November 2003 to October 2004	IFA	2.25%	Alexandre 2006
473 dogs	Bragança	Blood	-	IFA and conventional or nested PCR	12.7% (IFA) and 0% (PCR) positive for *B. burgdorferi* s. l.	Figueiredo 2007
649 cats from veterinary medical centers and shelters	Lisbon, Setúbal and Faro	Blood	January 2012 to August 2013	Nested PCR	2.2% Positive for *B. burgdorferi* s. l.	Maia et al. 2014b
593 ticks collected from 283 hosts (25 different species)	Castelo Branco, Portalegre, Lisbon, Setúbal, Beja and Faro	Ticks	December 2013 to September 2015	PCR	0%	Pereira et al. 2018
2915 ticks collected from vegetation	Braga, Vila Real, Aveiro, Lisbon, Setúbal, Évora and Faro	Ticks	May 2012 to May 2014	Nested PCR	3.3% positive for *B. burgdorferi* s. l. from Braga, Vila Real, Lisboa, Setúbal, Évora and Faro. Of this percentage, 24% matched with *B. lusitaniae* and *B. garinii*, 11% with *B. burgdorferi* s. s., 10% *B. valaisiana* and 3% *B. afzelli*	Nunes et al. 2016
300 ticks from vegetation	Madeira island	Ticks	-	Nested PCR	2.7% positive ticks for *B. lusitaniae*	Carvalho et al. 2008
5535 ticks	Nation scale, Mafra and Grândola	Ticks	March-July 2001 to March-July 2002 (Nation wide); March 1999 to December 2002 (Mafra) and April 1999 to February 2003 (Grândola)	Culture and nested PCR	14.2% positive for *Borrelia* spp.	Baptista et al. 2004
371 ticks from vegetation	Alentejo	Ticks	December 2006 to April 2009	Nested PCR	18.3% positive for *B. lusitoniae*	Milhano et al. 2010
925 ticks from dogs, cats and vegetation	Guarda, Lisbon, Setúbal and Faro	Ticks	May 2012 to May 2013 (cats and dogs) and June 2012 (vegetation)	Nested PCR	0.1% positive for *B. burdorferi* s. l.	Maia et al. 2014a
654 ticks from vegetation	Madeira Island	Ticks	2004	Nested PCR	0% positive for *B. burgdorferi* s. l.	Dietrich et al. 2010

Legend: CVBD-Canine Vector-borne diseases; PCR- Polymerase chain reaction; IFA- Indirect Immunofluorescence

Regarding dog infections, Alho et al. (2016), detected for the first time *B. afzelli* DNA in dogs in Portugal (3.2%), which is one of the most causative agents of tick-borne disease in Europe and North America. Maia et al. (2015) detected the first molecular evidence of naturally occurring *B. burgdorferi* s. l. in dogs in Portugal (0.8%). These antibodies were detected in Algarve, Bragança, Alentejo, Lisbon, North and Center Portugal using serology (Alexandre 2006; Figueiredo 2007; Cardoso et al. 2012). To our knowledge, only one study by Maia et al. (2014b) reported the prevalence of *B. burgdorferi* s. l. in cats in Portugal (2.2%).

Evidence shows that LD spreads geographically due to climate change (Kilpatrick et al. 2017; Garrido and Borges-Costa 2018). In recent years, several researchers have observed a rise in the prevalence of tick-borne illnesses in colder regions. This can be attributed to the increase in atmospheric temperature, which may facilitate and/or hasten the development of ticks. As a result, these vector-borne diseases may spread more widely and have a more global distribution (Rossati 2017; Rocklöv and Dubrow 2020; Rupasinghe et al. 2022). Furthermore, global warming may affect not only the survival of ticks in higher latitudes but also the host population (e.g. bird migration and rodents) and human behaviour (e.g. urban sprawl), which increases the risk of spreading this disease (Kilpatrick et al. 2017; Littman et al. 2018; Rosà et al. 2018).

## Clinical presentation

Borreliosis is usually asymptomatic or has very non-specific clinical signs, making it a multisystem disease (Kassab et al. 2020; Milkovičová et al. 2023). According to Little et al. (2010), only 5% of infected dogs show clinical signs, which might be a reason to be underdiagnosed in this species.

Lyme arthritis is the most prevalent clinical sign of borreliosis in dogs, and it is usually present in young and large-breed dogs with an outdoor lifestyle (Littman et al. 2018).

Three stages of the disease can be identified in dogs: (1) early localized disease, right after the tick bite and inoculation of bacteria, when a discrete inflammatory reaction (erythema migrans) occurs on the skin, particularly around the bite; (2) early disseminated disease, characterized by disseminated erythema migrans and cranial nerve paralysis (facial nerve), meningitis and carditis; and (3) late disease, characterized by arthritis, encephalopathy, and acute polyneuropathy (Liu et al. 2019).

As previously stated, LD in dogs typically has non-specific clinical signs, which include pyrexia, lymphadenopathy, anorexia, myalgia, lethargy, lameness, diarrhea, conjunctivitis and arthritis (with the tarsus and carpus being the most usually affected joints). Less frequently, infected dogs can also show kidney failure associated with protein-losing nephropathy, myocarditis, and neurological signs with consequent behavioral changes (aggression, convulsions or facial paralysis) (Little et al. 2010; Chomel 2015; Littman et al. 2018; CAPC 2019; Galluzzo et al. 2020; ESCCAP 2023). Lyme nephritis is considered a rare condition (5–10% of cases) with a poor prognosis with fatal evolution in dogs (Borys et al. 2019).

It is unclear how LD manifests clinically in cats. Some authors state that cats are less predisposed to develop borreliosis because they are more efficient in removing ticks (Littman et al. 2018). Studies stated that experimentally infected cats did not show detectable clinical signs of disease (Lappin et al. 2015). Lobetti (2017) stated that experimentally infected cats show signs of arthritis and meningitis. However, in naturally infected cats, clinical manifestations are uncommon (ESCCAP 2023), though it is difficult to prove that clinical signs are associated with LD since Anaplasmosis has the same vector and similar clinical signs (Littman et al. 2018). There is one study that reported that one cat showed clinical signs of disease in the USA, such as lethargy, lameness, anorexia and ataxia and responded to treatment with doxycycline and another one in the UK that reports recurrent pyrexia in two cats (Shaw et al. 2005; Hoyt et al. 2018). Tørnqvist-Johnsen et al. (2020) stated that although Lyme carditis is rare, two cats positive for *B. burgdorferi* with serology and polymerase chain reaction (PCR) presented this condition. One of the cats also had a history of erythema migrans.

In humans, erythema migrans is the most common sign, affecting 70–80% of the patients (Schwartz et al. 2019). Erythema can be followed by other “flu-like symptoms” such as fever, headaches, myalgia, arthralgia, and fatigue. The infection can spread to other tissues and organs (nervous system, joints and skin) (Ebani et al. 2014; Chomel 2015; Stanek and Strle 2018).

## Diagnosis

LD is difficult to diagnose because the clinical signs are non-specific, and subclinical infection may be present. Diagnosis should be made through a combination of tests (detection of specific antibodies and/or detection of *Borrelia* DNA), clinical signs, epidemiological factors, history/duration of exposure to ticks, exclusion of other diseases and response to antibiotics (Borys et al. 2019; CAPC 2019; Milkovičová et al. 2023).

Various diagnostic methods are available for LD, each with different sensitivity, specificity, and practical aspects (Table 2). Serology is considered the primary method of confirmation when LD is suspected (Kassab et al. 2020). Antibodies against this agent appear 3–5 weeks after infection and can be detected with commercial immunochromatographic tests (ESCCAP 2023). However, during the first few weeks of infection, a meager immune response can lead to false negatives (Johnson 2011). The most popular tests include IFA, ELISA and the Western Blot test. Vaccines can create antibodies that become detectable in IFA or ELISA tests, and previous exposure to the agent can also lead to false positives. The ELISA test has moderate sensitivity and specificity, allowing for the processing of multiple samples simultaneously (ESCCAP 2023 and Liu et al. 2023). However, it cannot distinguish between active infections and prior exposure or vaccination. In contrast, the Western Blot test is highly sensitive and specific, serving as a confirmatory test following a positive ELISA result. It is particularly useful in differentiating between antibodies produced due to vaccination and those from natural infection (ESCCAP 2023 and Liu et al. 2023). Western-Blot, is considered the gold standard, as it differentiates positive animals. Moreover, there are currently commercial kits that detect antibodies against the C6 synthetic peptide derived from the IR6 region within the *Borrelia* membrane protein VLsE, not interfering with vaccine-derived antibodies (e.g. SNAP4DXPlus IDEXX) (Littman et al. 2018). This test can also detect antibodies in cats, and it’s validated for this species because a species-cross conjugate is used (Levy et al. 2003). The SNAP 4Dx Test offers moderate sensitivity and specificity as well. It provides results within minutes and is capable of detecting antibodies to *Borrelia burgdorferi* and other tick-borne pathogens, making it useful for initial screening purposes (ESCCAP 2023 and Liu et al. 2023).

There are other methods of diagnosing LD, such as culture, microscopy and PCR (Table 2). However, they may not have a high sensitivity (ESCCAP 2023). *Borrelia burgdorferi* spirochetes are almost impossible to detect in blood smears by microscopy or Gram staining. They can be found in the soft tissues after the infection and are only temporarily in the blood. In these cases, dark field or phase contrast immunofluorescence microscopy is more accurate (Cutler et al. 2017; WOAH 2020). Observing spirochetes in synovial fluid also has low sensitivity (CAPC 2019; ESCCAP 2023). Culture is also not a highly sensitive method due to the low density and distribution of bacteria found in chronic infections (CAPC 2019; Milkovičová et al. 2023). Also, culturing *Borrelia* spp., may take longer than two weeks to grow and is particularly challenging. Therefore, it is neither practical nor timely to use culturing to diagnose chronic infections (Replogle et al. 2021).

PCR allows the amplification of the agent’s DNA from the dog’s fluids, tissue and tick fragments. In cats, the best material for PCR is synovial membrane, skin samples or renal biopsies. This test helps diagnose LD, and real-time quantitative PCR has improved pathogen diagnosis over the last decade (Straubinger 2000). Some authors referred to PCR as a sensitive method that can detect low quantities of organisms (Borchers et al. 2015). Therefore, it is important to note that a negative result in a PCR test does not necessarily rule out the presence of *Borrelia* spp. (Bergmann and Hartmann 2017). This is because the number of spirochetes in infected tissues or body fluids is often deficient. Additionally, the proper procedures for sample collection, transport, and DNA extraction are critical for obtaining reliable and consistent PCR results (Wang et al. 2010). Furthermore, the sensitivity of PCR detection varies depending on the type of specimens and the timing of collection (Marques 2015). The PCR test has variable sensitivity but high specificity. It is useful for detecting active infections and confirming diagnoses in ambiguous cases. However, its effectiveness can be limited by transient bacteremia or difficulties with sampling, such as when using synovial fluid ESCCAP 2023 and Liu et al. 2023). Furthermore, the LAMP (Loop-Mediated Isothermal Amplification) test exhibits high sensitivity and specificity and is simpler and faster than PCR. It holds potential for point-of-care testing due to its quick turnaround time and minimal equipment requirements, although it is still under research (ESCCAP 2023 and Liu et al. 2023). Lastly, Next-Generation Sequencing (NGS) provides very high sensitivity and specificity, offering detailed genetic information about strain variations. However, it is mainly used in research settings due to its complexity and cost (ESCCAP 2023 and Liu et al. 2023).

**Table 2:** Summary of diagnostic methods of Lyme Disease (ESCCAP 2023 and Liu et al. 2023)

Test	Sensitivity	Specificity	Practical aspects
ELISA	Moderate	Moderate	Ability to process multiple samples. Cannot distinguish active infection from prior exposure/vaccination.
Western Blot	High	High	Used as a confirmatory test following a positive ELISA result; differentiates between antibodies from vaccination and natural infection.
SNAP 4Dx Test	Moderate	Moderate	Provides results within minutes; detecting antibodies to *B. burgdorferi* and other tick-borne pathogens, useful for initial screening.
Culture	Low	Low	*Borrelia burgdorferi* may take longer than two weeks to grow and is particularly challenging. Not practical.
PCR	Variable	High	Useful for detecting active infection and confirming diagnosis in ambiguous cases; limited by transient bacteremia or sampling difficulties (e.g. synovial fluid).
LAMP	High	High	Simpler and faster than PCR. Potential for point-of-care testing due to short time and minimal equipment needs; Still under research.
NGS	Very High	Very High	Provides detailed genetic information about the strain variation; Used mainly in research due to complexity and cost.

Legend: ELISA - Enzyme-Linked Immunosorbent Assay; LAMP - Loop-Mediated Isothermal Amplification; PCR- polymerase chain reaction; NGS - Next-Generation Sequencing

## Treatment and prevention

The two main pillars of treatment can be summarized as solving the bacterial infection and managing the pain (arthritis) (Littman et al. 2018). Doxycycline (at a dose of 10 mg/kg PO or IV, SID or BID for a minimum of 1 month) is considered the first choice antibiotic. Other antibiotics in the class of tetracyclines, penicillins, macrolides and cephalosporins are also considered suitable for treatment (Littman et al. 2018; ESCCAP 2023). According to Littman et al. (2018), treatment with doxycycline is likely to be effective in cats.

In a dog with acute arthritis, antibiotic treatment should show some improvements within one to three days (Littman et al. 2018). Analgesics, such as gabapentin, non-steroidal anti-inflammatory drugs or glucocorticoids, may also be administered in the presence of neuropathic pain (Littman et al. 2018). When protein-losing nephropathy is present, diuretics, ACE inhibitors, dietary management, anticoagulants and fluid therapy can also be administered (Littman et al. 2018).

**Fig. 2:**
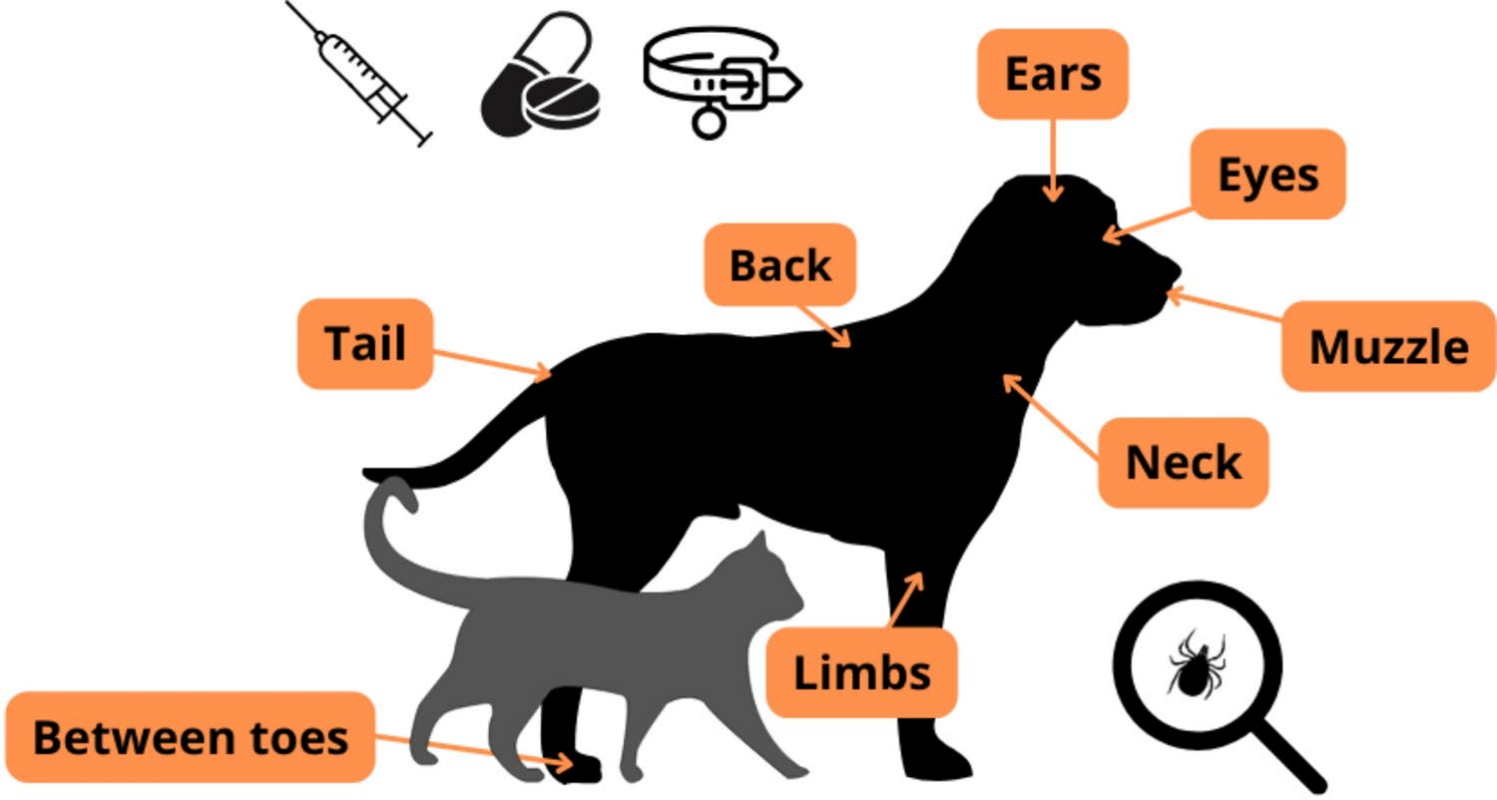
Methods to prevent LD in companion animals. Where to perform physically check for ticks

The preventative methods have been summarized in Table 3; Fig. 2. Tick control has become the method of choice (Bergmann and Hartmann 2017; Qurollo 2019; ESCCAP 2023). There are many formulations for tick control, such as collars, *spot-ons* and oral formulations. (Seresto^®^) collars (imidacloprid 10% + flumethrin 4,5%) showed to be effective for at least seven months in preventing diseases caused by the tick bite in 100% of the dogs studied by Krämer et al. (2020). Sarolaner (SimparicaⓇ) and afoxolaner (NexguardⓇ) also prevent tick infections (Six et al. 2016). It is important to note that topical permethrins should not be applied to cats (Littman et al. 2018).

**Table 3:** Methods to prevent LD in companion animals

Method	Practical aspects
Lyme Disease Vaccine	Administered to dogs in endemic areas as a preventive measure; Debate over long-term efficacy.
Tick Preventive Products (Topical)	Monthly application of products with active principles to kill or repel ticks; Neurological or allergic reactions rarely.
Tick Preventive Products (Oral)	Oral medications given monthly or every three months; Neurological or allergic reactions rarely.
Regular Tick Checks	Physically check pets for ticks after they have been outdoors, particularly in endemic areas; remove ticks promptly to prevent disease transmission; Time consuming and ineffective.

In addition to the use of effective ectoparasiticides, inspecting and brushing the animals’ coats in the areas where ticks have excellent affinity (limbs, ears, muzzle and corners of the eyes), cutting vegetation or grass in the pets’ habitat and rodent control can be measures to control ticks and also wild animals that are infested with ticks (CAPC 2019; WOAH 2020; Nascimento and Barros 2023).

Some vaccines are also available for dogs to control *B. burgdorferi*, but their efficacy is still unclear, and some only protect against *B. burgdorferi* s. s. (Littman et al. 2018; Stillman et al. 2019; ESCCAP 2023).

Advisory boards encourage all dogs getting veterinary care to undergo a yearly screening for vector-borne diseases to promote early diagnosis, treatment, and prevention (Creevy et al. 2019).

## Conclusion

LD has been a concern in Europe for several decades due to change in its distribution and prevalence. The spread of this disease and others to new areas is influenced by traveling and migration of people and animals, climate change, and the importation of animals from endemic areas. Close contact between animals and humans increases the risk of transmitting pathogenic diseases such as LD. Thus, it is important to perform diagnostic tests whenever there is a history of being exposed to ticks and/or showing clinical symptoms compatible with the disease. It is also important to educate pet owners about the significance of tick control in dogs and cats to prevent the transmission of tick-borne diseases.

Therefore, One Health and transdisciplinary approach is crucial to reducing the incidence of this zoonosis. Cooperation between veterinarians, medical doctors, public health agents, and other health professionals is crucial to prevent, monitor and manage future cases and outbreaks of LD in different hosts.

## Data Availability

No datasets were generated or analysed during the current study.

## Abbreviations

BID: Bis in die (twice a day)
CAPC: Companion Animal Parasite Council
CDC: Centers for Disease Control and Prevention
CVBD: Canine Vector-borne diseases
ECDC: European Centre for Disease Prevention and Control
ELISA: Enzyme-Linked Immunosorbent Assay
ESCCAP: European Scientific Counsel Companion Animal Parasites
IFA: Indirect Immunofluorescence
INE: Institudo Nacional de Estatística
IV: Intravenous
LAMP: Loop-Mediated Isothermal Amplification
NGS: Next-Generation Sequencing
PCR: polymerase chain reaction
PO: Per os (orally)
SID: Semel in die (once a day)
UK: United Kingdom
USA: United States of America
WHO: World Health Organization
WOAH: World Organization for Animal Health
